# Small Bowel Metastasis of Osteosarcoma Presenting with Intussusception: A Case Report

**DOI:** 10.70352/scrj.cr.26-0161

**Published:** 2026-06-13

**Authors:** Wataru Otsu, Yuki Shitomi, Shigeo Ninomiya, Wataru Miyoshino, Michihiro Ichimanda, Yoshimasa Aoyama, Takahiro Hiratsuka, Tomonori Akagi, Tomotaka Shibata, Hidefumi Shiroshita, Masafumi Inomata

**Affiliations:** 1 Department of Gastroenterological and Pediatric Surgery, Oita University Faculty of Medicine, Yufu, Oita, Japan; 2 Department of Comprehensive Surgery for Community Medicine, Oita University Faculty of Medicine, Yufu, Oita, Japan; 3 Advanced Emergency and Critical Care Center, Oita University Faculty of Medicine, Yufu, Oita, Japan; 4 Department of Advanced Medical Personnel Nurturing, Oita University Faculty of Medicine, Yufu, Oita, Japan

**Keywords:** osteosarcoma, small bowel metastasis, intussusception

## Abstract

**INTRODUCTION:**

Osteosarcoma is the most common primary malignant bone tumor in children and young adults. The lung is the most frequent site of metastasis, whereas extrapulmonary metastases, including those to the gastrointestinal tract, are rare. We report a case of small bowel metastasis of osteosarcoma presenting with intussusception.

**CASE PRESENTATION:**

A 19-year-old man with osteosarcoma of the left femur and synchronous pulmonary metastases underwent wide resection of the primary lesion followed by multiple pulmonary resections and multimodal chemotherapy, achieving temporary remission. Three years after the initial diagnosis, he presented with persistent fatigue and intermittent abdominal pain. Laboratory evaluation revealed severe anemia. Contrast-enhanced CT demonstrated intussusception of the proximal jejunum caused by an intraluminal tumor. Conservative treatment was unsuccessful, and surgical intervention was performed. Laparoscopic exploration revealed jejunal intussusception with a tumor serving as the lead point, which was confirmed at mini-laparotomy. Partial resection of the small bowel was undertaken. Histopathological examination showed atypical tumor cells with chondroid differentiation and osteoid formation, consistent with metastatic osteosarcoma. The postoperative course was uneventful, and the patient remained alive at 4 months after surgery. A review of the literature identified only 11 reported cases of small bowel metastasis from osteosarcoma, most of which presented with intussusception and were associated with a poor prognosis.

**CONCLUSIONS:**

Small bowel metastasis of osteosarcoma is extremely rare but should be considered in patients with a history of osteosarcoma presenting with intussusception. Surgical resection may provide symptomatic relief and plays an important role not only in definitive diagnosis but also in improving QOL.

## Abbreviation

CECT
: contrast-enhanced CT

## INTRODUCTION

Osteosarcoma is the most common primary malignant bone tumor in children and young adults and is characterized by a generally poor prognosis and a high incidence of pulmonary metastases.^1)^ According to previous reports, the lung is the most frequent site of metastasis (83.8%), followed by bone (21.9%), brain (2.9%), and liver (1.9%).^2)^ Metastasis to the gastrointestinal tract, particularly to the small bowel, is rare. However, with recent advances in multimodal therapy, including chemotherapy and surgical resection, survival outcomes have improved, resulting in an increasing number of reported cases of metastases to extrapulmonary organs.

When small bowel metastasis occurs, it may present with bleeding, perforation, obstruction, or, rarely, intussusception caused by an intraluminal tumor acting as a lead point. Among metastatic tumors, melanoma and lung cancer are the most common primary origins of small bowel intussusception.^3,4)^ In general, adult intussusception is an uncommon condition, accounting for approximately 5% of all intussusception cases and 1%–5% of intestinal obstruction in adults, and is frequently associated with an underlying neoplastic lesion. Against this background, intussusception secondary to metastatic osteosarcoma is exceedingly rare.

We report a rare case of metastatic osteosarcoma to the small bowel presenting with intussusception and discuss its clinical features in comparison with previously reported cases.

## CASE PRESENTATION

A 19-year-old man presented with persistent fatigue and intermittent abdominal pain lasting for 1 week. Three years earlier, he had been diagnosed with osteosarcoma of the left femur accompanied by synchronous pulmonary metastases. He underwent wide resection of the primary femoral lesion with tumor prosthetic reconstruction, followed by multiple pulmonary resections for recurrent metastatic disease. Specifically, 2 years and 4 months prior to the current presentation, he underwent right lower lobectomy with additional partial resections for 6 metastatic lung lesions, as well as partial resection of the left lung for 11 metastatic lesions. Following surgical management, he received multimodal chemotherapy consisting of methotrexate (MTX) and doxorubicin plus cisplatin (AP), followed by gemcitabine–docetaxel (GD) therapy, and subsequently achieved remission. He later presented to our hospital with persistent fatigue and intermittent abdominal pain. On physical examination, his vital signs were within normal limits. The abdomen was flat with mild firmness and tenderness noted in the mid-abdomen. Laboratory findings revealed severe anemia with a hemoglobin level of 5.3 g/dL. The C-reactive protein level was 2.29 mg/dL, and the white blood cell count was 6.97 × 10^3^/μL. CECT revealed a 50-mm lobulated mass in the proximal jejunum. The lesion served as the lead point of intussusception. Dilatation of the proximal small intestine was observed, with intraluminal contents suggestive of hemorrhage (**Fig. 1**). No definite high-density area suggestive of ossification was identified within the tumor. The lesion showed relatively homogeneous attenuation with gradual contrast enhancement. A small amount of ascites was present in the pelvis. There was no evidence of liver metastasis, and no new lesions were detected in the left femur or lungs. Conservative management was initially attempted; however, as no improvement was observed, surgical intervention was undertaken. Surgery was performed under general anesthesia. Laparoscopic exploration revealed intussuscepted small bowel. The procedure was then converted to a mini-laparotomy, and the intussuscepted segment was exteriorized (**Fig. 2**). The intussusception was reduced using the Hutchinson maneuver. A tumor was located approximately 30 cm distal to the ligament of Treitz, and approximately 15 cm of the small intestine had invaginated. Although the intussuscepted segment was dilated and edematous, no discoloration suggestive of ischemia was observed. Partial small bowel resection including the tumor was performed without systematic lymph node dissection, followed by functional end-to-end anastomosis. Gross examination revealed a 50 × 50 mm protruding (Type 1) tumor within the intestinal lumen. The tumor appeared segmented within the intestinal cavity (**Fig. 3**). Histopathological examination demonstrated solid proliferation of atypical round-to-spindle-shaped tumor cells with enlarged hyperchromatic nuclei and a high nuclear-to-cytoplasmic ratio (**Fig. 4A** and **4B**). Foci of chondroid differentiation (**Fig. 4C**) and osteoid formation (**Fig. 4D**) were observed. The histological features were consistent with those of the primary tumor, leading to the diagnosis of small bowel metastasis of osteosarcoma. All resection margins were histologically negative. Genomic profiling of the tumor was not performed. The postoperative course was uneventful. The patient was discharged on POD 8. Postoperative histopathological examination confirmed complete resection of the small bowel lesion, and the patient was subsequently managed with careful follow-up without additional adjuvant therapy. He remains alive 4 months after surgery without abdominal symptoms.

**Fig. 1:**
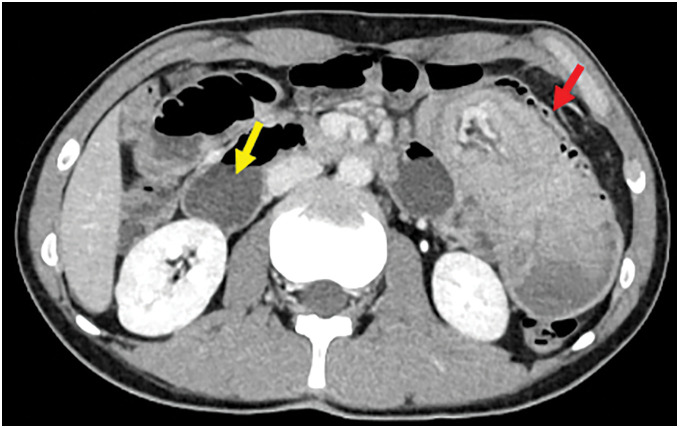
CECT showing a lobulated mass measuring 50 mm in the proximal jejunum (red arrow). The proximal small intestine is dilated (yellow arrow). CECT, contrast-enhanced CT

**Fig. 2:**
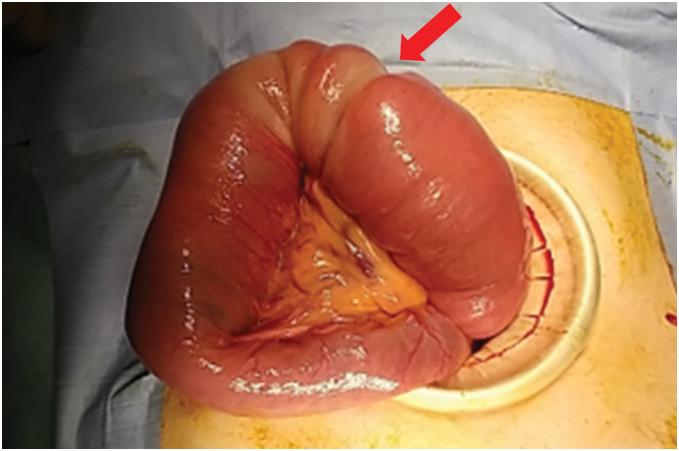
Intraoperative findings at mini-laparotomy showing jejunal intussusception located approximately 30 cm distal to the ligament of Treitz (red arrow).

**Fig. 3:**
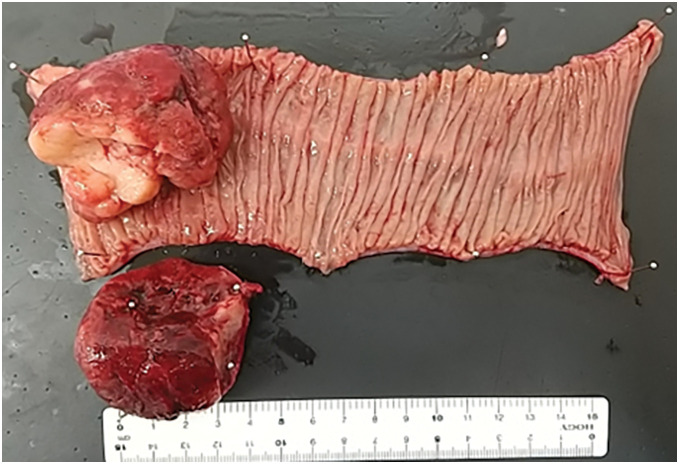
Gross specimen showing a 50-mm elastic, firm, whitish tumor protruding into the intestinal lumen. The apparent separate lesions represent different portions of the same tumor. The apparent separation occurred because the tumor was disrupted during specimen preparation.

**Fig. 4:**
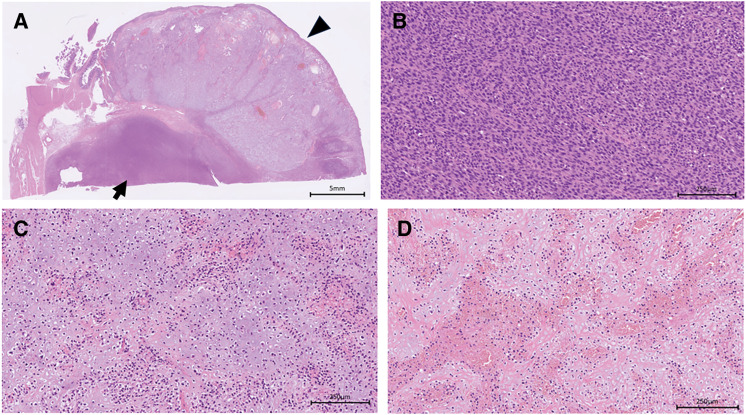
Histopathological findings. (**A**) Two distinct components were identified: solid tumor components (arrow) and areas showing chondroid differentiation and osteoid formation (arrowhead). (**B**) Tumor cells with oval-to-spindle-shaped nuclei proliferating in a solid pattern (×100). (**C**) Chondroid differentiation is observed (×100). (**D**) Focal osteoid formation is observed (×100).

## DISCUSSION

Although pulmonary metastasis is the predominant pattern of spread in osteosarcoma, involvement of the small bowel is exceedingly uncommon, and intussusception may be the initial manifestation. To the best of our knowledge, only 11 cases, including the present case, have been reported in the literature to date (**Table 1**).^5–14)^ As shown in **Table 1**, small bowel metastases from osteosarcoma have been reported to occur most frequently in the jejunum, and the present case is consistent with this pattern. The predominance of jejunal involvement among cases of small bowel metastasis may suggest the presence of an underlying mechanism, which warrants further investigation in future studies.

**Table 1:** Previously reported cases of small bowel metastasis of osteosarcoma

No.	Year	Age	Sex	Time to small bowel metastasis (months)	Metastasis site	Intussusception	Outcome
1	1987^5)^	29	F	60	Jejunum	+	N/A
2	1988^6)^	21	M	42	Jejunum	+	Death (2 months)
3	1999^7)^	42	F	120	Jejunum	+	N/A
4	2000^8)^	21	M	42	Jejunum, Ileum	–	N/A
5	2001^9)^	13	M	52	Jejunum	+	Death (4 days)
6	2003^10)^	9	M	24	Jejunum	+	N/A
7	2003^11)^	20	M	60	Jejunum	+	N/A
8	2007^12)^	58	F	19	Jejunum	+	Death (11months)
9	2016^13)^	23	F	12	Ileum	–	N/A
10	2024^14)^	43	M	156	Jejunum	–	N/A
11	2025*	19	M	36	Jejunum	+	Alive (4 months)

*Present case.

F, female; M, male; N/A, not applicable

Although follow-up strategies for osteosarcoma are generally similar to those for other high-grade malignancies, prolonged surveillance may be required due to the potential for late recurrence. Based on **Table 1**, the interval from the initial diagnosis of osteosarcoma to the detection of small bowel metastasis ranged from 12 to 156 months (median, 42 months), indicating that metastasis can occur even after a prolonged period. In addition, although routine abdominal surveillance is not standard, clinicians should be aware of the possibility of gastrointestinal metastasis and consider further evaluation when patients present with abdominal symptoms. Further accumulation of cases is needed to establish appropriate long-term follow-up strategies for osteosarcoma patients. The mechanism by which osteosarcoma metastasizes to the small bowel remains unclear. Panizo-Santos et al.^8)^ suggested hematogenous spread based on the presence of tumor cells within small arteries and veins. Similarly, Horiuchi and colleagues^12)^ reported that hematogenous metastasis was likely because the tumor was located in the submucosal layer and showed expansive growth. In our case, gross examination revealed a 50-mm elastic, firm, whitish tumor protruding into the intestinal lumen; however, the precise mechanism of metastasis remains uncertain.

Although CECT allows reliable diagnosis of intussusception, definitive qualitative diagnosis of the underlying cause is often difficult.^15)^ Consequently, many cases are diagnosed definitively only after surgical resection. In patients with a history of osteosarcoma presenting with intussusception, gastrointestinal metastasis, although rare, should be considered in the differential diagnosis. In the present case, intussusception was successfully diagnosed by CECT; however, as no calcification was observed, qualitative diagnosis of the tumor was difficult. Furthermore, the presence of intussusception made it difficult to perform small bowel endoscopy or other diagnostic examinations to establish a definitive diagnosis. A partial resection of the small intestine containing the tumor was performed as emergency surgery, and postoperative histopathological examination led to the diagnosis of small bowel metastasis of osteosarcoma. Systematic lymph node dissection was not performed in this case because the surgery was performed on an emergency basis and the definite diagnosis of the lesion had not been established preoperatively. Further advances in diagnostic modalities are warranted.

Based on previously reported cases, intussusception was present at diagnosis in 8 of the 11 reported cases (**Table 1**). Metastatic small bowel tumors typically exhibit an intraluminal protruding growth pattern,^15)^ which predisposes them to serve as a lead point due to intestinal peristalsis. In the present case, histopathological examination demonstrated chondroid differentiation and osteoid formation. Tumors with ossification, such as metastatic osteosarcoma, may form relatively firm and mobile masses because of their bone and osteoid components. These morphological features, in addition to the intraluminal growth pattern, may further increase the risk of intussusception.

As shown in **Table 1**, among the reported cases in which prognosis was described, all 3 patients died within 1 year. Although our patient remains alive, the follow-up period is relatively short at 4 months. These findings suggest that small bowel metastasis from osteosarcoma represents advanced systemic disease and is associated with a generally poor prognosis. However, as discussed above, small bowel metastases frequently present with intussusception and, in some cases, with severe anemia as observed in our patient, making palliative surgical intervention necessary in many instances. Further accumulation of cases is required to clarify the postoperative prognosis after resection.

## CONCLUSIONS

Small bowel metastasis of osteosarcoma is extremely rare but should be considered in patients with a history of osteosarcoma presenting with intussusception. Surgical resection may provide symptomatic relief and plays an important role not only in definitive diagnosis but also in improving QOL.

## References

[1] Mirabello L, Troisi RJ, Savage SA. International osteosarcoma incidence patterns in children and adolescents, middle ages and elderly persons. Int J Cancer 2009; 125: 229–34. 19330840 10.1002/ijc.24320 PMC3048853

[2] Xu G, Wu H, Zhang Y, et al. Risk and prognostic factors for different organ metastasis in primary osteosarcoma: a large population-based analysis. Orthop Surg 2022; 14: 714–9. 35293677 10.1111/os.13243 PMC9002071

[3] Blecker D, Abraham S, Furth EE, et al. Melanoma in the gastrointestinal tract. Am J Gastroenterol 1999; 94: 3427–33. 10606298 10.1111/j.1572-0241.1999.01604.x

[4] Niu QG, Huang MH, Kong WQ, et al. Stage IV non-small cell lung cancer with multiple metastases to the small intestine leading to intussusception: A case report. World J Clin Cases 2024; 12: 5960–7. 39286383 10.12998/wjcc.v12.i26.5960 PMC11287517

[5] Webster VJ, Arons I. Intussusception secondary to osteogenic sarcoma metastasis. Int J Clin Pract 1987; 41: 628–9. 3479186

[6] Mozes M, Mozes G, Greiff M, et al. Metastatic osteogenic sarcoma of small intestine with intussusception. Isr J Med Sci 1988; 24: 426–8. 3165973

[7] Ganguli SN, Hamilton P, Hanna S, et al. Small bowel intussusception secondary to osteogenic sarcoma metastasis: case report. Can Assoc Radiol J 1999; 50: 170–2. 10405649

[8] Panizo-Santos A, Sola I, Lozano M, et al. Metastatic osteosarcoma presenting as a small-bowel polyp. A case report and review of the literature. Arch Pathol Lab Med 2000; 124: 1682–4. 11079025 10.5858/2000-124-1682-MOPAAS

[9] Hung GY, Chiou T, Hsieh YL, et al. Intestinal metastasis causing intussusception in a patient treated for osteosarcoma with history of multiple metastases: a case report. Jpn J Clin Oncol 2001; 31: 165–7. 11386464 10.1093/jjco/hye031

[10] Chandramohan K, Somanathan T, Kusumakumary P, et al. Metastatic osteosarcoma causing intussusception. J Pediatr Surg 2003; 38: E1–3. 10.1016/s0022-3468(03)00517-7 14577092

[11] Wootton-Gorges SL, Stein-Wexler R, West DC. Metastatic osteosarcoma to the small bowel with resultant intussusception: a case report and review of the literature. Pediatr Radiol 2003; 33: 890–2. 13680015 10.1007/s00247-003-1018-2

[12] Horiuchi A, Watanabe Y, Yoshida M, et al. Metastatic osteosarcoma in the jejunum with intussusception: report of a case. Surg Today 2007; 37: 440–2. 17468831 10.1007/s00595-006-3414-3

[13] Badiu DC, Manea CA, Porojan V, et al. A rare cause of bowel obstruction: peritoneal metastases in osteosarcoma at the tibia in a young female patient with brain metastasis. Chirurgia (Bucur) 2016; 111: 274–8. 27452942

[14] Harne PS, Salooja I, Thomas P, et al. Gone to guts: osteosarcoma with metastasis to small and large intestines presenting as recurrent bleeding. ACG Case Rep J 2024; 11: e01224. 38192610 10.14309/crj.0000000000001224 PMC10773834

[15] Azar T, Berger DL. Adult intussusception. Ann Surg 1997; 226: 134–8. 9296505 10.1097/00000658-199708000-00003 PMC1190946

